# Acute Inflammatory Arthropathy and Hypercalcemia Leading to a Diagnosis of Primary Hyperparathyroidism in a Patient With Known Sarcoidosis

**DOI:** 10.7759/cureus.41110

**Published:** 2023-06-28

**Authors:** Kalee Larsen, Jenna Guma, Rime Mehannek, Michael Guma

**Affiliations:** 1 Internal Medicine, New York Medical College at Saint Michael's Medical Center, Newark, USA; 2 Internal Medicine, Cooper University Hospital, Camden, USA; 3 Internal Medecine, New York Medical College at Saint Michael's Medical Center, Newark, USA; 4 Internal Medicine/Rheumatology, New York Medical College at Saint Michael's Medical Center, Newark, USA; 5 Internal Medicine/Rheumatology, Hackensack University Medical Center, Hackensack, USA

**Keywords:** hypercalcemia, chondrocalcinosis, sarcoidosis, crystal arthropathy, inflammatory arthropathy, acute joint pain, hyperparathyroidism, pseudo-gout, calcium pyrophosphate deposition disease (cppd)

## Abstract

Calcium pyrophosphate deposition disease (CPPD) is a crystal-induced arthropathy characterized by calcium pyrophosphate crystal deposition in joints and soft tissues. The diagnosis is suggested by the presence of chondrocalcinosis on x-ray but is most often diagnosed by synovial fluid analysis (SFA). CPPD is associated with aging and metabolic disorders such as hyperparathyroidism. In this case, we present an 87-year-old woman with known sarcoidosis who presented with acute arthropathy, hypercalcemia, and radiographic evidence of CPPD. Her hypercalcemia had been attributed to her sarcoidosis in the past without a full workup. Hypercalcemia in the setting of suspected CPPD led to a full workup for hypercalcemia and ultimately led to a diagnosis of primary hyperparathyroidism. This case highlights the importance of a complete evaluation for hypercalcemia in the setting of CPPD, even when another disease, such as sarcoidosis, could explain hypercalcemia. Ultimately, CPPD aided in diagnosing hyperparathyroidism in our patient with known sarcoidosis.

## Introduction

Calcium pyrophosphate deposition disease (CPPD) is a common form of crystal-induced arthropathy characterized by calcium pyrophosphate (CPP) crystal deposition in joints and soft tissues, which leads to inflammation and joint damage. CPPD is associated with aging, hyperparathyroidism, hemochromatosis, hypophosphatemia, and hypomagnesemia. The diagnosis is often made by synovial fluid microscopic analysis (SFA); however, a negative SFA does not exclude the diagnosis [1-4]. When SFA is negative and clinical presentation is suggestive of CPPD, radiography can help make the diagnosis [4]. Diagnosing CPPD may be challenging, and it is crucial to have a high clinical suspicion for the disease. The diagnosis is essential as it may unearth associated metabolic disorders. In this case report, we present a case of CPPD and hypercalcemia in a patient with sarcoidosis, leading to the diagnosis of primary hyperparathyroidism. The co-occurrence of sarcoidosis and primary hyperparathyroidism is uncommon. This case highlights the importance of a thorough investigation and a high clinical suspicion for CPPD and its underlying causes.

## Case presentation

An 87-year-old female with a past medical history of sarcoidosis, hypertension, and a prior stroke presented to the emergency room with chest pain for two days. The pain was on the left anterior chest wall with no specific trigger. She had no other symptoms at the time of admission. Her sarcoidosis was diagnosed by lung biopsy many years ago, and she received intermittent steroids for pulmonary symptoms, mainly cough, for the past 40 years. Her last steroid treatment was nine months prior. Home medications included losartan 50 mg daily, esomeprazole 40 mg daily, and ranolazine 500 mg twice daily. She had never smoked and denied alcohol or illicit substance use. Her electrocardiogram and troponin were grossly normal, and her chest pain resolved. While in the hospital, she developed a painful, swollen knee and a fever of 102 Fahrenheit for three days. Rheumatology was consulted.

On evaluation by rheumatology, her temperature was 100 Fahrenheit, blood pressure was 132/65, pulse was 92, respiratory rate was 18, and saturation was 97% on room air. On physical examination, the patient was awake and alert. There was no parotid enlargement, no scleral erythema, and no adenopathy. The cardiopulmonary exam was unremarkable. There was mild tenderness to palpation of the left chest wall. The right knee was swollen, erythematous, warm, and tender to palpation. She had chronic lower extremity edema that was worse on the right compared with the left. There was no rash. Distal pulses were intact. Cranial nerves were grossly intact. Strength and sensation were intact in her bilateral upper and lower extremities. Initial serum labs and SFA are shown in Table 1. Labs were significant for hypercalcemia and SFA revealed cloudy fluid with 20,500 white blood cells, no crystals, and negative culture and gram stains.

**Table 1:** Initial serum labs, rheumatologic labs, and synovial fluid analysis BUN, blood urea nitrogen; AST, aspartate aminotransferase; ALT, alanine transaminase; ALP, alkaline phosphatase; WBC, white blood cell; MCV, mean corpuscular volume; BNP, brain or B-type natriuretic peptide; PTH, parathyroid hormone; ANA, antinuclear antibody; ELISA, enzyme-linked immunosorbent assay; RBC, red blood cell

Lab	Value	Normal range
Serum labs		
Sodium	138 mmol/L	136-145 mmol/L
Potassium	4.1 mmol/L	3.5-5.1 mmol/L
Chloride	103 mmol/L	98-107 mmol/L
Bicarbonate	24 mmol/L	22-29 mmol/L
BUN	20 mg/dL	10-20 mg/dL
Creatinine	0.9 mg/dL	0.3-1.5 mg/dL
Glucose	106 mg/dL	82-115 mg/dL
AST	19 U/L	5-34 U/L
ALT	11 U/L	0-55 U/L
ALP	88 U/L	40-150 U/L
Magnesium	2 mg/dL	1.6-2.6 mg/dL
Phosphorus	2.3 mg/dL	2.3-4.7 mg/dL
Calcium	12.9 mg/dL	8.4-10.2 mg/dL
WBC	5.8×10^3^/uL	4-11×10^3^/uL
Hemoglobin	10.2 g/dL	12-15.5 g/dL
Hematocrit	30.2%	36-46%
MCV	86.5 fL	80-100 fL
Platelets	246×10^3^/uL	135-430×10^3^/uL
Troponin	0.02 ng/mL	0.000-0.02 ng/mL
BNP	21 pg/mL	0-100 pg/mL
PTH	175 pg/mL	12-65 pg/mL
25-hydroxy-vitamin D	26 ng/mL	20-80 ng/mL
Rheumatologic labs		
ANA	Positive	Negative
ANA titer	1:40	<1:40
ANA pattern	Nuclear, homogenous	
Rheumatoid factor	15 IU/mL	<14 IU/mL
Anti-cyclic citrullinated peptide antibody	Negative	Negative
Lyme antibody	Negative	Negative
Lyme IgM ELISA	Negative	Negative
Lyme IgG ELISA	Negative	Negative
Synovial fluid analysis		
Fluid appearance	Cloudy	Clear
Color	Bloody	Colorless
RBC	28,600 cells/mm^3^	0-5 cells/mm^3^
WBC	20,500 cells/mm^3^	0-5 cells/mm^3^
Segmented neutrophils	89%	0-25%
Lymphocytes	11%	0-25%
Crystals	No crystals identified	No crystals
Gram stain	Negative	Negative
Culture	Negative	Negative

**Figure 1:**
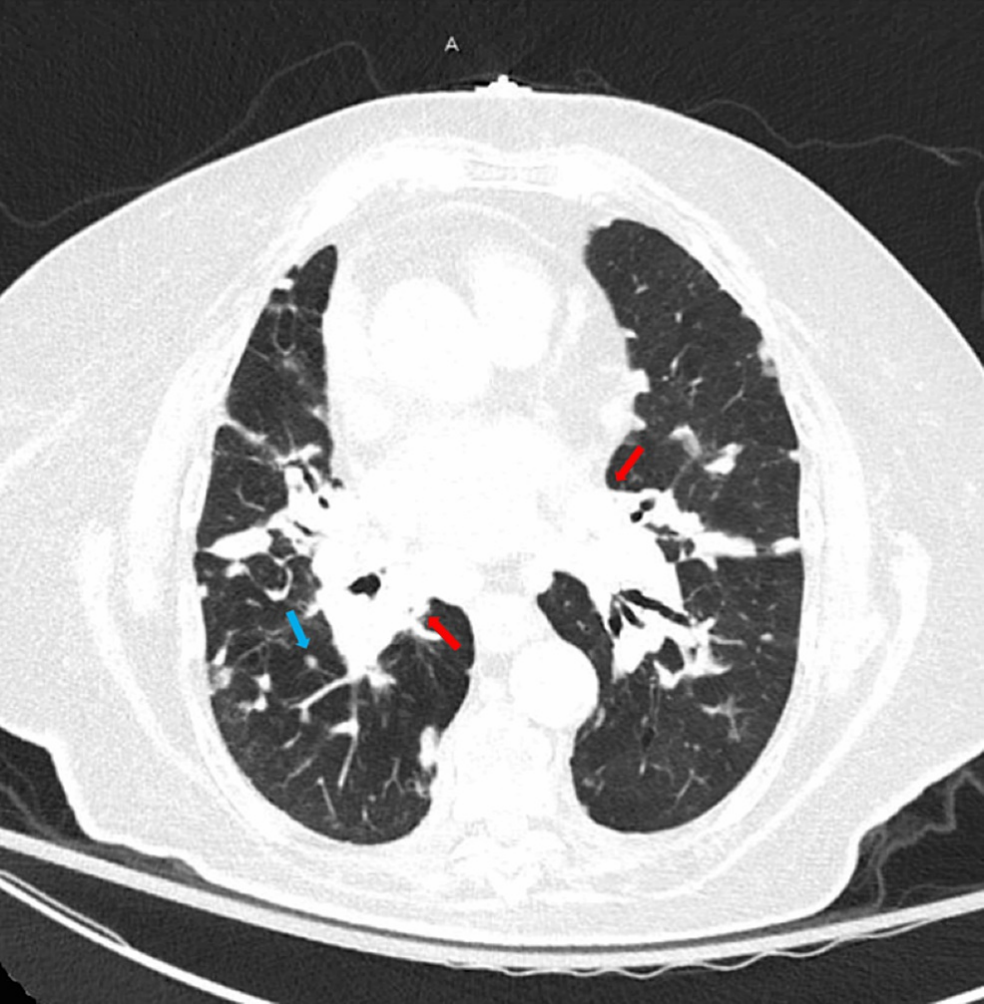
Chest computed tomography without contrast showing moderate peribranchial vascular nodularity (blue arrow) and extensive calcified mediastinal and bilateral hilar adenopathy (red arrows) unchanged from previous imaging and consistent with stable sarcoidosis

**Figure 2:**
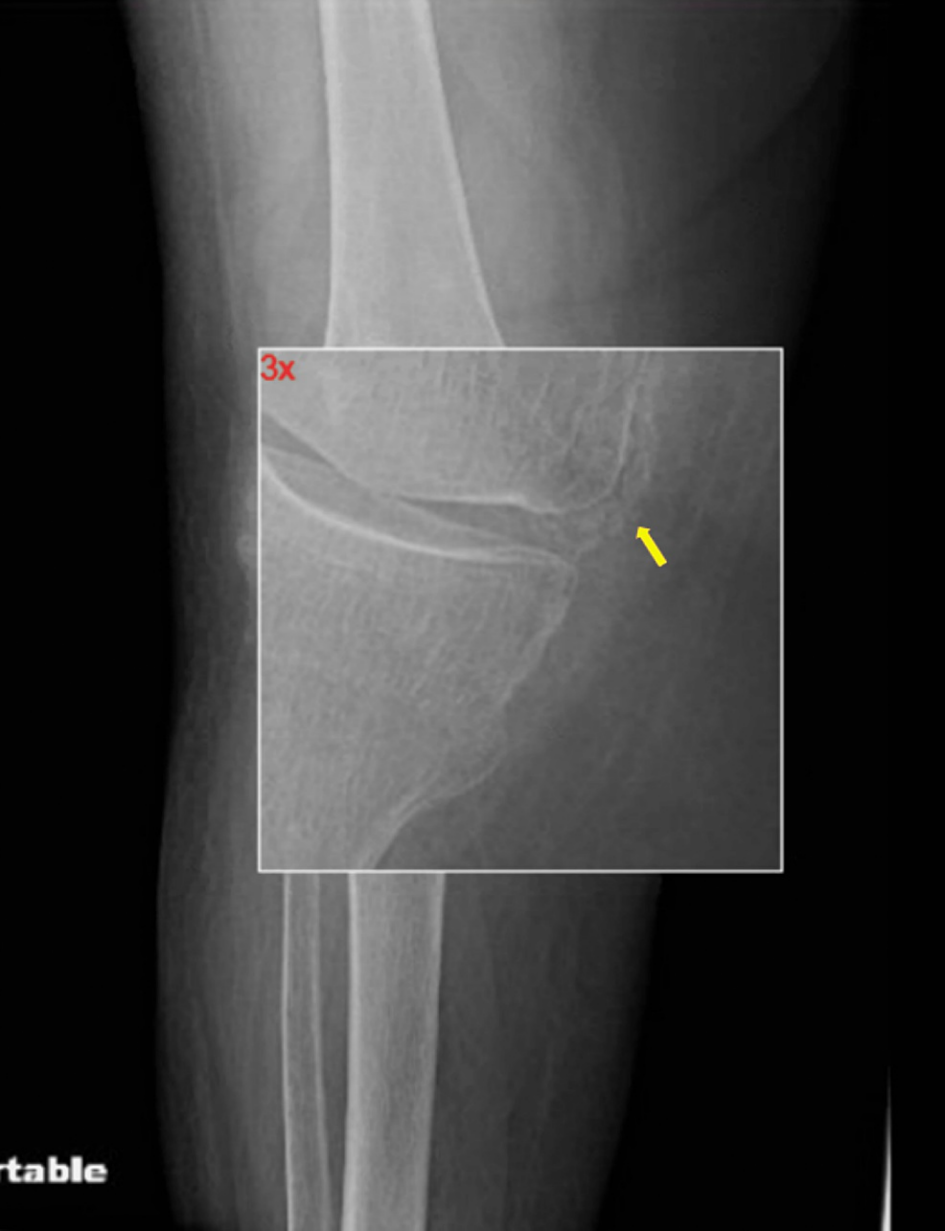
Magnified view of portable x-ray of the right knee showing chondrocalcinosis (yellow arrow)

Chest computed tomography without contrast showed moderate peribronchial vascular nodularity and extensive calcified mediastinal and bilateral hilar adenopathy unchanged from previous imaging, consistent with stable sarcoidosis (Figure 1). X-ray of the right knee revealed thinning of the bones, joint space narrowing, osteophytes, and chondrocalcinosis (Figure 2).

There was a high clinical suspicion for CPPD considering the clinical presentation, evidence of chondrocalcinosis on knee x-ray, and SFA suggestive of inflammatory arthropathy. Her hypercalcemia in the setting of suspected CPPD led to a workup for hyperparathyroidism. Vitamin D level and parathyroid hormone (PTH) were ordered (Table 1). The elevated PTH supported our suspicion of primary hyperparathyroidism associated with CPPD. The patient was treated with 20 mg of intravenous methylprednisolone for three days while inpatient. She responded in the first 24 hours to corticosteroid treatment with defervescence and resolution of her knee pain. She was discharged on a gradual oral prednisone taper over two weeks with instructions to follow up with rheumatology and endocrinology for her hyperparathyroidism. She was lost to follow-up after discharge from the hospital.

## Discussion

This case highlights the importance of a thorough investigation of metabolic disorders associated with CPPD. Our patient had a longstanding history of sarcoidosis and her hypercalcemia had been overlooked as a sequela of sarcoidosis. Sarcoidosis was in our differential for acute arthropathy; however, her presentation would have been atypical. Usually, acute sarcoid arthropathy presents as part of Lofgren’s syndrome, characterized by the triad of bilateral hilar adenopathy, migratory polyarthralgia, and erythema nodosum. Radiographic findings include cystic or sclerotic lesions and a lacy pattern of multiple lesions [5-8]. Our patient’s symptoms were not typical of sarcoid arthropathy; her clinical presentation, inflammatory arthropathy on SFA, and chondrocalcinosis on knee imaging were suggestive of CPPD despite SFA that was negative for crystals.

CPPD is characterized by CPP crystal deposition in joints and soft tissues, leading to inflammation and joint damage. The disease most commonly affects the knees, hips, ankles, shoulders, elbows, wrists, and toes and usually presents in older adults. CPPD is often idiopathic and is related to aging but is also associated with metabolic disorders such as hyperparathyroidism, hemochromatosis, hypophosphatemia, and hypomagnesemia [1,3]. The four sub-types of CPPD include asymptomatic presentation; acute CPP crystal arthritis, which is usually mono-articular and mimics gout but is less severe; chronic CPP crystal inflammatory arthritis, which produces polyarthritis and mimics rheumatoid arthritis; and osteoarthritis with CPPD, which occurs in joints not commonly associated with osteoarthritis, such as the metacarpophalangeal joints [1,4].

The gold standard for diagnosis of CPPD is SFA, which yields parallelepipedic, predominantly intracellular crystals with absent or weak positive birefringence [1]. However, SFA has low reliability for detecting CPP crystals. When SFA is negative and the clinical presentation is suggestive of CPPD, radiography showing chondrocalcinosis of the affected joint helps to make the diagnosis [4]. Multiple conditions have correlations with CPPD; hyperparathyroidism presents the highest positive association, followed by hemochromatosis. For this reason, we screen patients for both comorbidities, but the yield is most remarkable in those less than 50 years old with polyarticular disease [2].

Our patient’s clinical presentation and knee x-ray were consistent with acute CPP crystal arthritis. Our patient was an octogenarian presenting with CPPD in one joint, typically associated with aging. However, the high serum calcium was suspicious and led to further investigation for associated metabolic disorders. In this case, CPPD aided in the diagnosis of primary hyperparathyroidism. Our patient’s hypercalcemia was likely secondary to both sarcoidosis and hyperparathyroidism. Hypercalcemia is present in sarcoidosis due to the activation of 1-alpha-hydroxylase by sarcoid granulomas leading to the conversion of 25-OH vitamin D to the active form of vitamin D,1,25-(OH)2 vitamin D. The active form of vitamin D increases gut calcium absorption and resorption of calcium from bones. In hyperparathyroidism, PTH promotes renal 1-alpha-hydroxylation of vitamin D and activation of osteoclasts leading to increased bone resorption [9-11].

## Conclusions

Our patient had a known history of sarcoidosis, and her hypercalcemia had been overlooked in the past. Her acute arthropathy and chondrocalcinosis suggested CPPD. Hypercalcemia in the setting of suspected CPPD led to further evaluation and diagnosis of primary hyperparathyroidism. This case illustrates the importance of a thorough investigation for metabolic disorders associated with CPPD, even when another disease may explain abnormalities found during the evaluation.
